# High throughput protein-protein interaction data: clues for the architecture of protein complexes

**DOI:** 10.1186/1477-5956-6-32

**Published:** 2008-11-26

**Authors:** James R Krycer, Chi Nam Ignatius Pang, Marc R Wilkins

**Affiliations:** 1 School of Biotechnology and Biomolecular Sciences, University of New South Wales, Sydney, Australia; 2 Systems Biology Initiative, School of Biotechnology and Biomolecular Sciences, University of New South Wales, Sydney, Australia

## Abstract

**Background:**

High-throughput techniques are becoming widely used to study protein-protein interactions and protein complexes on a proteome-wide scale. Here we have explored the potential of these techniques to accurately determine the constituent proteins of complexes and their architecture within the complex.

**Results:**

Two-dimensional representations of the 19S and 20S proteasome, mediator, and SAGA complexes were generated and overlaid with high quality pairwise interaction data, core-module-attachment classifications from affinity purifications of complexes and predicted domain-domain interactions. Pairwise interaction data could accurately determine the members of each complex, but was unexpectedly poor at deciphering the topology of proteins in complexes. Core and module data from affinity purification studies were less useful for accurately defining the member proteins of these complexes. However, these data gave strong information on the spatial proximity of many proteins. Predicted domain-domain interactions provided some insight into the topology of proteins within complexes, but was affected by a lack of available structural data for the co-activator complexes and the presence of shared domains in paralogous proteins.

**Conclusion:**

The constituent proteins of complexes are likely to be determined with accuracy by combining data from high-throughput techniques. The topology of some proteins in the complexes will be able to be clearly inferred. We finally suggest strategies that can be employed to use high throughput interaction data to define the membership and understand the architecture of proteins in novel complexes.

## Introduction

Most cellular processes involve multiprotein complexes [1-3]. Proteins interact either transiently or stably within these complexes, with identical or different subunits. Many proteins are also members of more than one complex, and thus part of a protein-protein interaction network inside the cell [2-4]. The interaction networks are highly dynamic, allowing for rapid changes in the proteome such as to external stimuli [4]. Despite the contribution of protein complexes and interactions to the regulation and execution of biological processes, relatively few complexes are well-understood in terms of structure and function [5].

High-throughput techniques, such as yeast-two hybrid (Y2H) [6] and affinity-purification/mass-spectrometry (AP-MS), have accelerated the generation of protein-protein interaction (PPI) data on a large scale. Following pioneering studies on the interactome [7], several large-scale studies have been undertaken in yeast and other species (e.g. [3,8-10]). These have led to the development of some high quality datasets of pairwise PPIs. For instance, the filtered yeast interactome (FYI) is an intersection of different datasets, including Y2H data, AP-MS data, *in silico *predictions, Munich Information Centre for Protein Sequences physical interactions, and protein complexes reported in the literature (see Han *et al*. [11] for details). It contains 2,493 high-confidence interactions for 1,379 proteins.

An alternative to studying the interactions of individual proteins is to define all complexes in the cell (the 'complexome') and their constituent proteins. In a study by Gavin *et al*. [12], tandem affinity purification tagging [13] was used to define 491 protein complexes in yeast, 257 of which were novel. Multiple replicate purifications revealed that within each complex, proteins could be classified as core, module, or attachment proteins, according to the frequency of their appearance in the various forms of that complex. Core proteins were present in most purifications of a complex, whilst attachment proteins were dynamic members present only some of the time. Module proteins were two or more attachment proteins, found together in more than one complex [14]. We have recently elucidated the structural basis of Gavin *et al*.'s classification [12], finding that interactions between core proteins and between two or more module proteins are likely to be mediated by domain-domain interactions. Interactions within and between attachment proteins were less likely to occur in this manner [14].

A novel avenue of investigation made possible with the Y2H technique has been to build low-resolution models of complexes. This is achieved by determining the subunit architecture of complexes and the manner in which subunits interact. The yeast yeast RNA polymerase (pol) III [15], ribonuclease P (RNase P) [16] and protein complexes involved in human mRNA degradation [17], for example, have been investigated by these means although in a focused, low-throughput way. Despite the steady accumulation of protein-protein interaction and protein complex data generated by large-scale screens, these data have not been widely used to understand the detailed architecture of protein complexes. A few studies have compared high throughput data to well-characterised complexes to define any limitations [2,18,19]. For instance, Edwards *et al*. [19] compared past interaction datasets to known three-dimensional structures of RNA polymerase II, Arp2/3, and the proteasome. They found that the interactions defined by individual high throughput methods were inconsistent with published information about these complexes. However, when integrated together, data from high throughput studies provided higher accuracy of interactions and greater insight into the structure of complexes [19]. Since this study, improved high-throughput datasets such as FYI [11] and Gavin *et al*.'s [12] data on the yeast complexome have been published. These have not been examined in detail to reveal their correlation with the architecture of known complexes nor have these data been examined to understand which can best define the members of a complex and provides clues to structural associations.

In this study, we have investigated the manner in which high throughput data or combinations of this data reflect the architecture of proteins in three large, well-defined complexes – the proteasome, the mediator and the SAGA complexes. We show that high throughput data concerning the interactions of individual proteins, particularly in combination, can accurately define the members of a protein complex. However, the same data were surprisingly poor in accurately predicting physical proximity of proteins. Data from HTP studies of protein complexes were weaker in accurately defining constituent subunits, but the core and module proteins were useful to help understand the architecture or topology of protein complexes.

## Methods

### Two-dimensional structural representation of protein complexes

Two-dimensional structural representations of protein complexes were generated, derived from structural or structure-associated data in the literature. A representation of the 20S core particle (CP) of the proteasome was derived from its X-ray crystal structure [20] As the structure of the yeast 19S regulatory particle (RP) has not been elucidated, a model of the 19S RP was adapted from Ferrell and coworkers [21], based on genetic and biochemical studies, and Sharon and colleagues [22] based on high range mass-spectrometry. A structural representation of the mediator complex was derived from models proposed using electron microscopy [23] and an interaction network based on genetic and biochemical data [24]. Med19 (Rox3) was recently shown to be part of the middle module (instead of the head module) [25], and this was taken into account in our representation. A structural representation of the SAGA complex was adapted from a model derived using electron microscopy, immunolabelling, and mutant complexes [26] This study proposed that the SAGA complex contained five modules (Domains I-V), and others have proposed that additional proteins are likely to be part of these modules [27] SAGA-associated proteins were obtained from Daniel and Grant [28].

### Protein-protein interaction datasets

Experimental data for protein-protein interactions were from several sources. Data on protein complexes was from Gavin *et al*. [12]. Complexes 2 and 75 described the 20S and 19S proteasomal subcomplexes respectively while Complexes 81 and 445 described the SAGA and mediator complexes respectively. Filtered yeast interactome (FYI) data was sourced from Han *et al *[11], and domain-domain interaction (DDI) data was extracted from iPfam release 20.0 [29] using custom Perl scripts, version 5.8.7, as described previously [14].

### Mapping interaction data onto the structures of protein complexes

To understand how data from different high-throughput analytical techniques can help accurately define the protein membership of complexes and elucidate topology, protein-protein interaction data was mapped onto our 2-D representations of the structures of complexes. Experimental pairwise interactions from the FYI dataset and DDI were represented by lines between protein nodes whilst membership of complexes, according to Gavin *et al *[12], was represented by node shading.

## Results and discussion

### Two-dimensional representations of protein complexes

**Figure 1:**
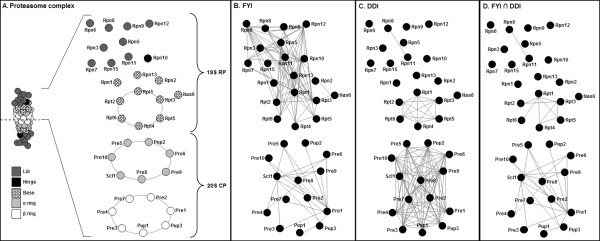
**Two-dimensional structural representations of the proteasome, overlaid with filtered yeast interactome (FYI) and predicted domain-domain interaction (DDI) data.** (A) The 2-D representation of the 19S RP is built from genetic, biochemical and mass-spectrometric data (see Methods) and the representation of the 20S CP is built from the known 3-D structure. Note that the proteasome is symmetrical and thus we show only the top half here. (B) Pairwise protein interaction data from FYI clearly groups proteins of the 19S RP and 20S CP into two separate groups but show interactions between non-adjacent proteins. (C) Predicted domain-domain interactions are seen between members of the proteasome base. They are also seen within and between the α and β rings of the 20S CP. (D) The intersection of FYI pairwise interactions and predicted domain-domain interactions.

**Figure 2:**
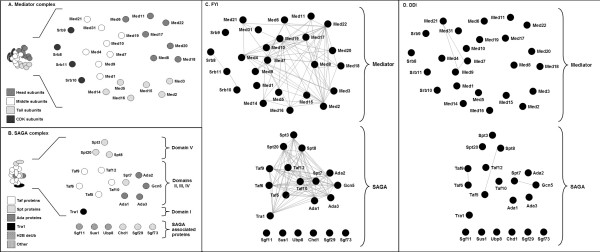
**Two-dimensional structural representations of the mediator and SAGA complexes, overlaid with filtered yeast interactome (FYI) and predicted domain-domain interaction (DDI) data.** (A) and (B) The 2-D representations of the mediator and SAGA complexes are built from structural and interaction data, but note that 3-D structures of these complexes are not known. (C) Pairwise protein interaction data from FYI clearly defined the mediator and SAGA complexes. Interactions between many structurally non-adjacent proteins, however, are seen. (D) Very few domain-domain interactions could be predicted for the mediator or SAGA complexes.

Our investigation involved analysing the proteasome and two transcriptional coactivator complexes. These were chosen as they are large, multisubunit complexes that have well-characterised structures. The proteasome complex, responsible for the degradation of most proteins in the cell, is composed of a 20S cylindrical core particle (CP) flanked by two 19S regulatory particles (RPs) each which contain a base and a lid [30]. The mediator complex passes information from gene-specific activators and repressors to core transcriptional machinery *via *its interaction with TFIIH and RNA polymerase II (RNAP II) [31,32]. It is comprised of 25 subunits, found in the head, middle, tail, and CDK modules [23,33]. The SAGA complex regulates transcription of stress-induced and highly-regulated genes *via *histone acetylation and direct interaction with the TATA-binding protein and other transcription factors [34,35] It has three distinct functional modules [26,28,35,36] along with other associated proteins. We constructed 2-D representations of the proteasome (Figure 1A), mediator complex (Figure 2A) and the SAGA complex (Figure 2B) according to the Materials and Methods. Note that our representation of the proteasome considers just one half of the structure as the proteasome is symmetrical. We sought to understand explore two key issues for these complexes. Firstly, whether high throughput protein-protein interaction datasets could accurately determine the protein members of the complexes and secondly, whether pairwise protein interactions, those from protein complexes or those predicted from the presence of certain domains can provide clues to the topology or architecture of a complex.

### Can pairwise interactions clearly define protein complexes?

The FYI dataset is an intersection of different interaction datasets (see Han *et al *[11] for details), and is enriched for high confidence, pairwise protein interactions. We investigated how these pairwise interactions, when mapped as lines connecting proteins on our 2-D representations, reflected the features of candidate complexes. For the proteasome, interactions in the FYI dataset clearly defined the 19S RP as one complex and the 20S CP as an independent complex (Figure 1B). Protein membership was also very clear, with 100% of subunits showing at least one interaction with other subunits in the same complex. Proteins in the 19S RP show a large number of interactions with other proteins in the same complex, but far fewer interactions were seen between members of the 20S CP. It was noted, however, that the subcomplexes in the proteasome, for instance the 19S RP lid and base, could not be discerned from this data. The SAGA and mediator complexes were also clearly defined by the FYI data. All proteins of domains I to V of the SAGA complex showed multiple interactions, with most proteins showing evidence of interaction with every other protein in the complex (Figure 2C). However, it was striking that the SAGA-associated proteins showed no interactions with proteins in the SAGA complex itself; this may be due to these interactions being transient or perhaps refractory to analysis using one or more interaction-measuring techniques. In the mediator complex, proteins showed a large range in the number of their interactions, with some proteins showing 1 interaction but others showing >10. Interestingly, the CDK module subunits and protein Med1 showed no interactions with the rest of the mediator complex (Figure 2C). The CDK module is known to be a temporary inhibitor of the mediator, thus a lack of interactions may reflect the transient nature of its interactions with the mediator [32]. For the complexes examined here, it was apparent that high quality pairwise interactions, from the FYI database, could accurately indicate whether proteins are likely to form a protein complex and which proteins are the constituent subunits.

### Can high throughput analyses of complexes define their constituent proteins?

**Figure 3:**
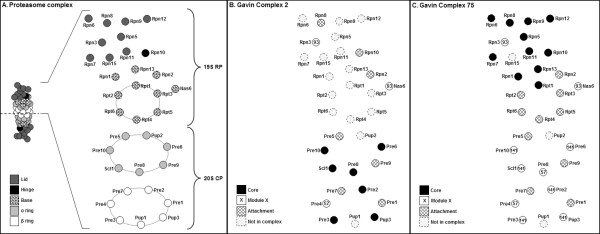
**Two-dimensional structural representation of the proteasome 19S RP and 20S CP, overlaid with high-throughput data from affinity purified protein complexes.** (A) 2-D representation as in Figure 1A. (B) Core, module and attachment proteins from Complex 2 [12] overlaid on the 2-D representation. Note that the core proteins are mostly those in the 20S CP. (C) Core, module and attachment proteins from Complex 75 [12] overlaid on the 2-D representation. Note that the core proteins here are mostly those in the proteasome lid and hinge.

**Figure 4:**
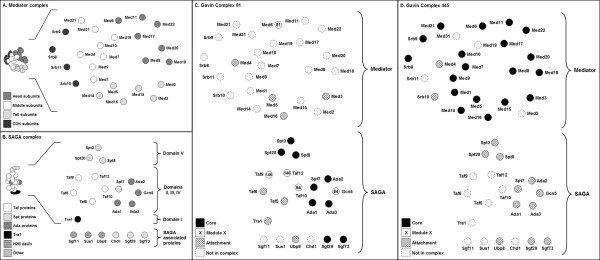
**Two-dimensional structural representations of the SAGA and mediator complexes, overlaid with high-throughput data from affinity purified complexes.** (A) and (B) 2-D representations as in Figures 2A and 2B. (C) Core, module and attachment proteins from Complex 81 [12] overlaid on the 2-D representation. Core proteins correspond to the Spt and Ada proteins in the complex. (D) Core, module and attachment proteins from Complex 445 [12] overlaid on the 2-D representation. Core proteins correspond to almost all proteins in the mediator complex.

To understand if data from high throughput AP-MS of complexes could accurately define the members of protein complexes, we mapped complexes from Gavin *et al*. [12] onto our 2-D representations. For the proteasome, the 19S and 20S subcomplexes corresponded to Gavin *et al*. [12] complexes 2 and 75 respectively. Encouragingly, a total of 91% of the known proteasomal subunits were seen in the high-throughput-defined complexes (see shading, Figures 3B,C). Similarly the Gavin *et al*. [12] complexes 81 and 445, which correspond to the SAGA and mediator complexes respectively, showed 85% of SAGA and 80% of mediator subunits (see shading, Figures 4C,D). This indicates that AP-MS has a high true positive identification rate for the constituent proteins of these complexes. However, it was also seen that high throughput AP-MS suggested a large number of other, false positive proteins as members of these complexes [see Additional files 1, 2, 3]. An additional 10 non-proteasome proteins (29%) and 23 non-mediator/SAGA proteins were observed (49%). This highlights that high throughput AP-MS alone may not accurately define the members of a complex, and is weaker than data from the FYI database for the clear definition of complexes and membership thereof. This contrasts with expectations that AP-MS, which seeks to purify protein complexes to homogeneity, should provide unambiguous data in this regard. It also contrasts with comments elsewhere [2,18,19] to this effect.

### Pairwise interactions do not accurately reflect the architecture of complexes

We next sought to understand the degree to which protein interaction data, represented as pairwise interactions in the FYI dataset, could provide clues into the architecture or topology of proteins in complexes. We examined whether FYI pairwise interactions were likely or indeed possible, by reference to our structural representations. In the proteasome 19S CP, the FYI data described interactions between many proteins that are known to be structurally associated (e.g. those that form the rings in the base such as Rpt4 and Rpt5, see Figure 1A,B). However a very large number of interactions were seen for many proteins that are unlikely to be structurally associated (e.g. the FYI data suggests Rpn12 to have >10 interactions, many of which are with proteins that are a considerable distance away in 3-D space, Figure 1B). In the proteasome 20S CP, there were fewer pairwise interactions documented, and many of these reflected structural associations (Figure 1B). For example, Pup3 is known to be adjacent to Pup1 and Pre1 in the β ring and proximal to Pre8 in the neighbouring α ring (Figure 1A) and this was seen in the FYI protein-protein interactions. However, the Pre1 protein showed 10 interactions, many of which were not consistent with the structural topology of the complex. For the SAGA and mediator complexes, a similar trend was observed. The FYI data described interactions between some proteins in the SAGA or mediator complexes that are structurally adjacent, however many proteins showed an excessively large number of interactions that were not consistent with the likely positions of protein subunits in 3-D space.

There are a number of possible explanations for FYI data being weak in reflecting the structural topology of protein complexes. Yeast two-hybrid experiments, which are a key part of the FYI dataset, are known to generate false positive interactions. These can arise due to the overexpression of proteins as part of the technique, their requirement to interact in the nucleus [18] and the possible involvement of one or more endogenous subunits that bridge the 'gap' between bait and prey proteins. This bridging could explain why, along with others, Gcn5 and Spt3 apparently interact in the SAGA complex when these subunits are spatially isolated [26] (Figure 2B). Yeast-two hybrid may also detect interactions mediated by homo-domain-domain interactions that may not normally occur (see later evaluation of domain-domain interactions); this may explain some of the unexpected interactions in the proteasome 20S CP. This is an important consideration, albeit usually ignored, in the interpretation of pairwise interactions in the FYI database.

### Core and module proteins reflect the architecture of a complex

From their high throughput screen of the complexome, Gavin *et al*. [12] proposed that complexes can have core proteins, present all the time, as well as module and attachment proteins which interact less strongly and/or only in certain conditions. To understand the relationship of core, module and attachment proteins to the architecture of protein complexes, we overlaid all protein types from Complexes 2, 75, 81, and 445 onto our 2-D representations (Figures 3, 4). Data is also given in tabular form in Additional file 2. In our analysis, we have ignored attachment proteins; this is because they are 'singletons' that were seen to occasionally interact with the cores of complexes [12]. They thus provided no information concerning the topology of a complex.

Core proteins in the proteasome, mediator and SAGA complexes (black shading, Figures 3B,C and 4C,D) were clearly seen to be interacting proteins in these complexes, particularly for the mediator complex, the proteasome 19S RP and 20S CP. It should be noted that whilst all 20S CP core proteins were not adjacent in our 2-D representations, they do in fact interact as part of the stacked α and β rings [37] For the SAGA complex, the core proteins were seen in three groups. Interestingly, the SAGA complex is yet to be crystallised and our model was built by considering data from electron microscopy, immunolabelling studies and mutant complexes [26-28,38] The core protein data suggests these 3 protein groups are almost certainly physically associated in the topology of the SAGA complex. Thus, for the proteins examined here and others in the Gavin *et al*. [12] dataset, core proteins are likely to be spatially grouped together and define aspects of the topology of complexes.

Modules, representing two or more proteins, were defined as those that are sometimes associated with the core of a complex [12]. It was expected that they reflect topological features of the complexes of interest. For the proteasome, it was clear that modules 57 and 141 were comprised of proteins that were structurally associated, being adjacent or near-adjacent in our models (see numbered proteins, Figure 3C). However module 93, comprised of proteins Rpn3 and Nas6, was not consistent with our 2-D structural representation. Note that our model of the 19S RP was adapted from Ferrell and coworkers [21] and Sharon and colleagues [22], and was built in the absence of a 3-D crystal structure. Thus, it remains possible that either Rpn3 or Nas6 may be incorrectly positioned or that module 93 is not true. Evidence that Nas6 interacts with Rpn3 in the literature [29] and FYI (see Figure 1B), and FYI evidence that Rpn3 interacts with its expected neighbours (Figure 1B), suggests the latter may be the case. Examining the SAGA complex, two modules were seen – Modules 84, and 146 (see numbered proteins, Figure 4C). The proteins in Module 146 are adjacent in our model and are likely to be structurally associated; this is consistent with their classification as a module. The proteins of Module 84 (Gcn5 and Taf10), whilst proposed to both interact with Spt7 [26], are not yet known to interact directly. Their existence as a module provides some evidence that this may be the case. To summarise, modules described proteins that in many cases were physically associated in a complex. This provides useful clues to the topology of proteins in a quaternary structure.

### Predicted domain-domain interactions can identify structural features of a complex

Finally, predicted domain-domain interactions (DDIs) between proteins were mapped onto our 2-D representations of complexes. Domain-domain interactions are not high throughput data *per se*, but were examined to understand how this data type can help in the interpretation of high throughput protein-protein interactions. The predicted DDIs in the proteasome were numerous and reflected many aspects of the 3-D positions of proteins. All proteins in the 19S RP base showed domain-domain interactions with each other (see lines connecting proteins in Figure 1C). The proteins Rpt1-6 were predicted to interact with each other by a common AAA-ATPase domain (Pfam: PF00004). This is structurally accurate as they interact with each other in a hexameric ring in the order Rpt1/2/6/4/5/3 [21,39]. However cross-ring DDIs were also predicted due to the presence of same domain, but are unlikely to occur as they are inconsistent with the proteaseome's 3-D structure. In the 20S CP, DDIs were seen within and between all proteins of the α and β rings (Figure 1C). Around-ring DDIs were seen in the 20S CP, as would be expected. Some incorrect cross-ring DDIs were also predicted, for example between Pre1 and Pre3 that are not adjacent proteins within or between the α and β rings [20,37] (Figure 1C). The reason why these were observed is due to the presence of the proteasome domain (Pfam: PF00227) in all 20S CP subunits. This domain is a putative homomeric interaction domain and thus each subunit could theoretically bind to the other subunits in the complex. Interestingly, this might explain some of the FYI interactions that were inconsistent with the proteasome topology (see Figure 1B); this is further highlighted by the overlap of many proteasome 20S CP DDIs with interactions documented in the FYI database (see Figure 1D).

In the co-activator complexes, only 6 domain-domain interactions were predicted (Figure 2D). Many of these, for example Med7-Med9 and Spt7-Gcn5, are between proteins that we expect to be structurally adjacent. In contrast to the proteasome, hetero-domain-domain interactions were seen to feature here. For example, Med7 and Med9 were predicted to interact *via *the Med7 protein domain (Pfam: PF05983) and RNA polymerase II transcription mediator domain (Pfam: PF07544). Part of the reason for this difference is that the interactions were extracted from the iPfam database, which is based on structural data. The structure of the proteasome is known from X-ray crystallography and mass spectrometry [22,37] whilst the structure of the SAGA and mediator complexes are mostly based on interaction studies [24] and electron microscopy [23,26]. Accordingly, the domain-domain interactions found for the co-activator exist only due to the observation of similar domain pairs between other proteins in crystallised complexes.

A comparison of predicted DDIs in the proteasome and the co-activator complexes suggests that where a complex contains many proteins that are paralogs or of similar domain content, such as complexes with ring structures, DDI interactions are unlikely to help understand the precise structural association of subunits within a complex. However, the accurate prediction of structural associations from DDI data may be possible in complexes that contain essentially unrelated proteins whose interactions are mediated by hetero-domain-domain interactions.

## Conclusion

In this study, we have generated 2-D structural representations of three well-characterised protein complexes. We compared high-throughput experimental data and DDI data against these 2-D representations to determine the degree to which the data reflect true structural associations of proteins. Whilst the 2-D representations, we believe, are useful means to approach this analysis it should be noted that we did not consider the stoichiometry within the complexes. Further, the 3-D structures of the co-activator complexes are inferred but unknown. Nevertheless, numerous interactions reflected structural features of the protein complexes and these are discussed below.

Complexes described by Gavin *et al*. [12] were useful for understanding structural associations of proteins in the proteasome, mediator and SAGA complexes. Core proteins in these complexes reflected the true interactions and associations of many proteins whilst module proteins captured small groups of proteins that are physically co-associated. Attachment proteins were not anticipated to provide strong insight into the structure of complexes and indeed many of these were false positive interactors or interactors that, due to weak or transient interaction, are yet to be conclusively associated with the complexes studied here.

The FYI dataset of pairwise interactions was the most useful and accurate means to determine membership of the proteasome, mediator and SAGA complexes. However these data did not provide clear insight into the structural topology of complexes due to an over-representation of false positive interactions. This is an important observation as the FYI dataset, which might be expected to have a reduced degree of false positives as any interaction needs to be seen at least twice [11], still overestimated the degree of true interactions. Interestingly, the more widespread use of iterative Y2H interactions as pioneered in Rain *et al*. [7] could address this issue in the future. Our study of predicted domain-domain interactions for the proteasome and co-activator complexes revealed a variable numbers of such interactions, being influenced by the lack of available structural data for the co-activator complexes and the presence of shared domains in paralogous proteasomal proteins. Thus whilst we have shown elsewhere[14] that DDIs can explain the mechanism of interaction of core and module proteins in Gavin complexes [12], the utility of DDIs to predict the 3-D topology of proteins in many complexes will require a far greater number of complexes to be studied with NMR or X-ray crystallography to better populate domain-domain interaction databases.

Having examined the insights that high throughput analyses can provide, we may ask: how can HTP data be used to help predict the topology of complexes in cases where complexes are not well characterised? Aloy *et al*. [40] explored this issue prior to the availability of protein core-module-attachment descriptions of complexes [12] and without reference to the FYI dataset [11,41], but managed to structurally model 42 complexes and partially model a further 12. We expect that use of these new resources in the following way would help expand on this, at least for the topology of complexes. Gavin *et al*. [12] complexes can be used as an initial template. An overlay of high quality pairwise interaction data, such as the FYI dataset [11,41], should be useful to eliminate spurious interactors and thus confirm protein membership of the complex. The core proteins can be considered as a group of structurally associated proteins and the modules as groups of proteins for which physical interaction (particularly for 2-protein modules) are highly likely. In many cases, the examination of domain-domain interactions in core and module proteins will assist in understanding the likely pairwise interactions that occur within the core and module of complexes; whilst this was not seen in the three complexes we have examined here, we have recently shown that this is the case for many Gavin-defined complexes in the yeast cell [14]. Finally, the resulting pairwise interaction data might be projected into a 3-D space using tools such as GEOMI [42] to construct a possible representation of the complex. The approach will be most accurate where high-quality data is available, although the lack of information from high-throughput analyses on the stoichiometry of proteins in each complex may complicate the resulting predictions.

## Abbreviations

2-D: two-dimensional; HTP: high throughput; Y2H: yeast two hybrid; FYI: filtered yeast interactome; PPI: protein-protein interaction; DDI: domain-domain interaction; SAGA: Spt-Ada-Gcn5-acetyltransferase; RP: regulatory particle; CP: core particle; TFIID: transcription factor II D; NMR: nuclear magnetic resonance.

## Competing interests

The authors declare that they have no competing interests.

## Authors' contributions

JRK built the 2-D representations of the structures, mapped data onto these, generated the figures in the manuscript and co-drafted the manuscript. CNIP generated and processed interaction data sets, generated and analysed domain-domain interaction data, drafted some sections of the manuscript and critically revised the document. MRW designed and directed the project, co-drafted and critically revised the manuscript. All authors read and approved the manuscript.

## Supplementary Material

Additional file 1 **Compilation of high-throughput data for the 19S RP and 20S CP proteasomal subunits, as well as other proteins in Complexes 2 and 75 in Gavin *****et al ***[12]. A table that illustrates the degree to which high throughput complexes reflect the proteins of the proteasome. Click here for file

Additional file 2 **Compilation of high-throughput data for proteins in the mediator and SAGA complexes and associated proteins described in Complex 81 and 445 in Gavin *et al*. **[12]. A table that illustrates how high throughput complexes reflect the protein subunits of the mediator and SAGA complexes. Click here for file

Additional file 3 **Classification of proteasomal and coactivator protein subunits from Gavin *****et al ***[12]. A table showing core, module and attachment classification of proteins in the proteasomal and coactivator protein complexes. Click here for file
