# Supplementary material for: High throughput protein-protein interaction data: clues for the architecture of protein complexes
Source: Proteome Sci. 2008 Nov 26;6:32. doi: 10.1186/1477-5956-6-32 (PMC2621150; doi:10.1186/1477-5956-6-32)
Supplement: Additional file 1 — Compilation of high-throughput data for the 19S RP and 20S CP proteasomal subunits, as well as other proteins in Complexes 2 and 75 in Gavin et al [12]. A table that illustrates the degree to which high throughput complexes reflect the proteins of the proteasome. [file 1477-5956-6-32-S1.pdf]

Additional File 1. Compilation of high-throughput data for the 19S RP and 20S CP proteasomal subunits, as well as other proteins in Complexes 2 and 75 in Gavin *et al.*[12]

Complex	Subcomplex	OLN	Gene Name	Gavin 2	Gavin 75
19S RP[a]	Base	YGR232W	*NAS6*	M(93)	M(93)
		YHR027C	*RPN1*	–	C
		YIL075C	*RPN2*	A	A
		YLR421C	*RPN13*	–	C
		YKL145W	*RPT1*	–	C
		YDL007W	*RPT2*	–	A
		YDR394W	*RPT3*	–	A
		YOR259C	*RPT4*	–	A
		YOR117W	*RPT5*	–	A
		YGL048C	*RPT6*	–	A
	Hinge	YHR200W	*RPN10*	A	C
	Lid	YER021W	*RPN3*	M(93)	M(93)
		YDL147W	*RPN5*	–	C
		YDL097C	*RPN6*	–	C
		YPR108W	*RPN7*	–	C
		YOR261C	*RPN8*	A	C
		YDR427W	*RPN9*	–	C
		YFR004W	*RPN11*	–	C
		YFR052W	*RPN12*	–	C
		YDR363W-A	*RPN15*	–	–
20S CP[b]	α ring	YMR314W	*PRE5*	A	A
		YOL038W	*PRE6*	C[c]	M(141)
		YML092C	*PRE8*	C[d]	M(57)
		YGR135W	*PRE9*	A	A
		YOR362C	*PRE10*	C[c]	M(141)
		YGR253C	*PUP2*	–	–
		YGL011C	*SCL1*	C[c]	M(141)
	β ring	YER012W	*PRE1*	A	A
		YPR103W	*PRE2*	C[c]	M(141)
		YJL001W	*PRE3*	C[c]	M(141)
		YFR050C	*PRE4*	M(57)	M(57)
		YBL041W	*PRE7*	A	A
		YOR157C	*PUP1*	–	–
		YER094C	*PUP3*	C[c]	M(141)
Other	Proteasome activator	YFL007W	*BLM3*	A	A
	Cell division control protein	YJL194W	*CDC6*	–	A
	Proteasome-associated protein	YHL030W	*ECM29*	A	C
	U3 snoRNP protein	YNL075W	*IMP4*	A	–
	DNA repair protein	YLR035C	*MLH2*	A	A
	DNA repair protein	YNL250W	*RAD50*	–	A
	Suppressor of Rho3	YPR032W	*SRO7*	–	A
	Deubiquitinating enzyme	YFR010W	*UBP6*	–	A
	Uncharacterised protein	YOL087C	*	–	A
	Uncharacterised protein	YOL098C	**	–	A

Within each subcomplex, the proteins have been listed alphabetically by gene name. For each protein, the ordered locus name (OLN), gene name, and classification in Gavin *et al.*[12] Complexes 2 and 75 ('Gavin 2' and 'Gavin 75' respectively) are provided. Gavin *et al.*[12] classified proteins as core proteins (C), attachment proteins (A), or into a module X (M(X)). Other proteins from Complexes 2 and 75 (that were not part of either proteasomal complex) have been included under 'Other', with a brief description of their function in the 'Subcomplex' column obtained from Swissprot (www.expasy.ch) and Schmidt *et al.*[39]

* (OLN YOL087C) and ** (OLN YOL098C) are novel proteins. The ribosomal protein Rps18b (OLN YML026C) was part of Complex 75 but was excluded from this table because ribosomal proteins are considered to be common, non specific contaminants that generate false positives.[5]

[a] The protein composition of the 19S RP was sourced from Schmidt *et al.*[39] and Walz *et al.*[30]. Since Nas6 interacts with the ATPase ring (in the 19S RP base) via Rpt3, it was assumed that Nas6 is also part of the 19S RP base.

[b] The protein composition of the 20S RP was sourced from Baumeister *et al.*[37]

[c] This protein was classified as both a core and Module 141 protein by Gavin *et al.*[12] in Complex 2.

[d] This protein was classified as both a core and Module 57 protein by Gavin *et al.*[12] in Complex 2.
