# Supplementary material for: High throughput protein-protein interaction data: clues for the architecture of protein complexes
Source: Proteome Sci. 2008 Nov 26;6:32. doi: 10.1186/1477-5956-6-32 (PMC2621150; doi:10.1186/1477-5956-6-32)
Supplement: Additional file 2 — Compilation of high-throughput data for proteins in the mediator and SAGA complexes and associated proteins described in Complex 81 and 445 in Gavin et al. [12]. A table that illustrates how high throughput complexes reflect the protein subunits of the mediator and SAGA complexes. [file 1477-5956-6-32-S2.pdf]

Additional File 2. Compilation of high-throughput data for proteins in the mediator and SAGA complexes and associated proteins described in Complex 81 and 445 in Gavin *et al.*[12]

Complex	Subcomplex	OLN	Gene Name	Gavin 81	Gavin 445
Mediator [a]	Head	YHR058C	*MED6*	M(85)	C[d]
		YBR193C	*MED8*	−	C
		YMR112C	*MED11*	−	C
		YER022W	*MED17*	−	C
		YGR104C	*MED18*	−	C
		YBL093C	*MED19*	−	–
		YHR041C	*MED20*	−	C
		YBR253W	*MED22*	−	C
	Middle	YPR070W	*MED1*	−	C
		YOR174W	*MED4*	A	A
		YOL135C	*MED7*	−	C
		YNR010W	*MED9*	−	C
		YPR168W	*MED10*	−	C
		YDR308C	*MED21*	−	C
		YGL127C	*MED31*	−	–
	Tail	YDL005C	*MED2*	−	–
		YGL025C	*MED3*	A	C
		YGL151W	*MED5*	A	C
		YLR071C	*MED14*	–	C
		YOL051W	*MED15*	−	C
		YNL236W	*MED16*	A	C
	CDK Module	YCR081W	*SRB8*	−	C
		YDR443C	*SRB9*	−	C
		YPL042C	*SRB10*	−	A
		YNL025C	*SRB11*	−	–
SAGA[b]	Domain I	YHR099W	*TRA1*	A	–
	Domains II, III, and IV	YPL254W	*ADA1*	C	A
		YDR448W	*ADA2*	C	A
		YDR176W	*ADA3*	C	A
		YGR252W	*GCN5*	M(84)	A
		YBR081C	*SPT7*	C	A
		YBR198C	*TAF5*	A	–
		YGL112C	*TAF6*	A	–
		YMR236W	*TAF9*[c]	M(146)	–
		YDR167W	*TAF10*[c]	M(84)	–
		YDR145W	*TAF12*	M(146)	–
	Domain V	YDR392W	*SPT3*	C	A
		YLR055C	*SPT8*	C	A
		YOL148C	*SPT20*	C	A
	Other[c]	YER164W	*CHD1*	–	–
		YPL047W	*SGF11*	−	−
		YCL010C	*SGF29*	C	A
		YGL066W	*SGF73*	C	A
		YBR111W-A	*SUS1*	–	–
		YMR223W	*UBP8*	A	A
Other	NuA4, INO80, SWR1 subunit	YJL081C	*ARP4*	A	−
	NuA4 complex subunit	YFL024C	*EPL1*	A	−
	NuA4 complex subunit	YOR244W	*ESA1*	A	−
	NuA4 complex subunit	YDR359C	*VID21*	A	A
	Type III PIP kinase	YFR019W	*FAB1*	−	A

Coproporphyrinogen III oxidase	YDR044W	*HEM13*	A	–
Myosin-like protein 1	YKR095W	*MLP1*	–	A
Mitochondrial ribosomal subunit	YHR147C	*MRPL6*	–	A
ATP-dependent helicase	YMR080C	*NAM7*	A	–
Transcription factor	YGL013C	*PDR1*	A	–
Serine/threonine kinase	YAL017W	*PSK1*	A	–
Rab guanine exchange factor	YNL272C	*SEC2*	A	–
SWI/SNF complex subunit	YNR023W	*SNF12*	A	A
TATA-box-binding protein	YER148W	*SPT15*	A	–
Heat shock protein	YBR169C	*SSE2*	–	A
TFIID subunit	YGR274C	*TAF1*	M(146)	–
TFIID subunit	YPL011C	*TAF3*	M(146)	–
TFIID subunit	YMR005W	*TAF4*	M(146)	–
TFIID subunit	YMR227C	*TAF7*	A	–
TFIID subunit	YML015C	*TAF11*	M(146)	–
Cell morphogenesis protein	YIL129C	*TAO3*	A	–
Valyl-tRNA synthetase	YGR094W	*VAS1*	A	–
Transcription factor	YML007W	*YAP1*	M(85)	M(85)

Within each subcomplex, the proteins have been listed alphabetically by gene name. For each protein, the ordered locus name (OLN), gene name, and its classification in Gavin *et al.*[12] Complex 81 and 445 (‘Gavin 81’ and ‘Gavin 445’ respectively) have been provided. Gavin *et al.*[12] classified proteins as core proteins (C), attachment proteins (A), or into a module X (M(X)). Other proteins from Complex 445 (that were not part of either coactivator complex) have also been included under ‘Other’, with a brief description of their function in the ‘Subcomplex’ column obtained from Swissprot (www.expasy.ch). The ribosomal proteins Rpl8a (OLN YHL033C), Rpl14a (OLN YKL006W), Rpl17b (OLN YJL177W), Rpl18b (OLN YOL120C), Rpl19b (OLN YBL027W), Rpl30 (OLN YGL030W), Rpl33a (OLN YPL143W), Rpl35b (OLN YDL136W), Rps14b (ONL YJL191W), and Rps17b (OLN YDR447C) were part of Complex 445, but were excluded from this table because ribosomal proteins are considered to be common, non-specific contaminants that generate false positives.[5] The proteins shaded in grey are also components of the NuA4 complex.

[a] The protein composition of the mediator was sourced from Chadick and Asturias[23] and Guglielmi *et al.*[24], with the exception that Med5 has recently been shown to be part of the tail subcomplex. Baidoobonso *et al.*[25] assignment of Med19 into the middle subcomplex was not taken into account in this table.

[b] The protein composition of the SAGA complex was sourced from [26,27] and [28] and the proteins were allocated to five domains as outlined by Timmers and Tora[27]. Domains II, III, and IV were combined together due to the shared subunits in these domains (specifically, Taf5 and Taf10 are found in Domains II and III, Taf6 and Taf9 are found in Domains II and IV, Ada1 and Taf12 are found in Domains III and IV).

[c] These proteins were defined as part of the SAGA complex after the allocation of domains[27].

[d] Med6 was classified as both a core and Module 85 protein by Gavin *et al.*[12]
