# Supplementary material for: High throughput protein-protein interaction data: clues for the architecture of protein complexes
Source: Proteome Sci. 2008 Nov 26;6:32. doi: 10.1186/1477-5956-6-32 (PMC2621150; doi:10.1186/1477-5956-6-32)
Supplement: Additional file 3 — Classification of proteasomal and coactivator protein subunits from Gavin et al [12]. A table showing core, module and attachment classification of proteins in the proteasomal and coactivator protein complexes. [file 1477-5956-6-32-S3.pdf]

Additional File 3. Classification of proteasomal and coactivator protein subunits from Gavin *et al.*[12].

Complex	Classification	Gavin *et al.* Complex	
Proteasomal complexes		**Gavin 2**	**Gavin 75**
19S RP (20)	Core	0.0 (0)	55 (11)
	Module		
	M(93)	10 (2)	10 (2)
	Attachment	15 (3)	30 (6)
	None	75 (15)	5.0 (1)
20S CP (14)	Core	50 (7)	0.0 (0)
	Module		
	M(57)	7.1 (1)	14 (2)
	M(141)	0.0 (0)	43 (6)
	Attachment	29 (4)	29 (4)
	None	14 (2)	14 (2)
Coactivator complexes		**Gavin 81**	**Gavin 445**
Mediator (25)	Core	0 (0)	76 (19)
	Module		
	M(85)	4 (1)	0 (0)
	Attachment	16 (4)	8 (2)
	None	80 (20)	16 (4)
SAGA (20)	Core	45 (9)	0.0 (0)
	Module		
	M(84)	10 (2)	0.0 (0)
	M(146)	10 (2)	0.0 (0)
	Attachment	20 (4)	55 (11)
	None	15 (3)	45 (9)

For each complex listed in the left-hand column, the distribution of its subunits between core, module, and attachment types are provided for the main complexes from Gavin *et al.*'s[12] dataset that are discussed in the text. The bracketed number after the complex name refers to the number of proteins found within that complex – for example, '19S RP (20)' equates to 20 proteins being found in the 19S regulatory particle of the proteasome. 'Gavin X' refers to the Complex X and M(Y) refers to Module Y, as defined by Gavin *et al.*[12]. The frequency of each classification type of protein is denoted by 'percentage (raw frequency)'. If a protein was classified as both a core and a module type within the same of one of Gavin *et al.*[12] complexes, then it was treated as a core protein during this analysis.
